# Using a Smartphone-Based Chatbot for Postoperative Care After Intravitreal Injection During the COVID-19 Pandemic: Retrospective Cohort Study

**DOI:** 10.2196/43022

**Published:** 2024-08-02

**Authors:** Pei-Chang Wu, Wei-Yu Chiang, Jung Lo, Jong-Jer Lee, Yung-Jen Chen, Hsi-Kung Kuo, Jie-Shin Chiau, Shu-Hui Hsu, Yi-Hao Chen

**Affiliations:** 1 Department of Ophthalmology Kaohsiung Chang Gung Memorial Hospital and Chang Gung University College of Medicine Kaohsiung Taiwan

**Keywords:** smartphone chatbot, postoperative care, intravitreal injection, age-related macular degeneration, self-report, endophthalmitis, COVID-19

## Abstract

**Background:**

During the COVID-19 pandemic period, it was difficult to carry out regular and scheduled follow-up of patients in the outpatient department, especially during lockdown periods. However, early detection of initial infection or other serious conditions is vital for patients after ocular surgery, such as intravitreal injection (IVI) for age-related macular degeneration (AMD).

**Objective:**

We evaluated the use of a smartphone-based postoperative care chatbot system (PCCS) with an instant bidirectional feedback system for patients to self-report postoperative symptoms and signs.

**Methods:**

During the COVID-19 level 3 epidemic alert in July 2021 in Taiwan, the PCCS alerted the patients to report and grade 6 ocular symptoms and signs associated with ocular inflammation or retinal detachment. Patients used the PCCS for 7 days post surgery to assess their symptoms and signs each day after receiving an alert. Data were automatically collected using a cloud computer system, including symptom grades and messages sent to medical staff for further medical assistance. A user satisfaction questionnaire was administered to the patients on the seventh day post surgery.

**Results:**

In total, 185 patients participated in this study. There were 26 (3.03%) reports of symptom grade deterioration (including increased blurred vision, eye swelling, nausea, and floaters or flashes) from 12 (6.5%) patients. We found no difference in the gender of patients who received an early medical consultation. One case of endophthalmitis was reported, wherein an improvement was observed after prompt administration of IVI antibiotics twice. Overall, 87% (n=185) of patients were satisfied or very satisfied with communicating their symptoms instantly through the app; they were willing to use it again and believed that it could improve the quality of care. Seven of the 185 (3.8%) patients had an earlier medical consultation and 1 (0.5%) had endophthalmitis.

**Conclusions:**

The chatbot system, designed for self-reporting postoperative symptoms and providing instant bidirectional feedback on smartphones, could be beneficial for enhancing the quality of care in early medical consultations without gender differences among patients with AMD receiving IVI, and achieved satisfactory responses from patients.

## Introduction

Age-related macular degeneration (AMD) is the leading cause of vision loss in patients older than 65 in the many countries [1]. Repeated intravitreal injection (IVI) of anti–vascular endothelial growth factor (VEGF) antibody is the gold standard and most common surgical treatment for AMD [2]. Clinical trials using monthly fixed doses have shown the best results, but findings are unavailable from real-world settings and are compromised owing to the use of less common strategies such as pro re nata and treat-and-extend [3,4]. Rare but serious postoperative complications leading to blindness may occur after IVI, such as endophthalmitis and retinal detachment [5,6]. The incidence of endophthalmitis after IVI is approximately 1 in 5000 patients (0.02%). Certain ocular signs and symptoms are associated with post-IVI ocular inflammation, including redness, tenderness, blurred vision, floaters, photopsia, or visual field defects. Early detection of associated symptoms and signs and prompt management could achieve better outcomes [7,8]. However, most patients receiving IVI of anti-VEGF antibody in the outpatient setting and in follow-up visits are ordinarily scheduled for assessment 1 month later to alleviate the follow-up burden resulting from a large number of injections [9]. There is a gap, which may delay treatment past the optimal window [10], especially during the COVID-19 pandemic. To support patients during this period, we developed a chatbot self-report system to provide an alternative way to monitor patients’ conditions.

In this study, we assessed the usability and feasibility of a smartphone-based postoperative care chatbot system (PCCS; Bipower Co) for assisting patients who received IVI. The PCCS periodically prompted patients to self-record and self-report post-IVI symptoms and signs and was equipped to send alerts automatically to medical staff for prompt feedback.

## Methods

### Overview

This retrospective cohort study was conducted at the Kaohsiung Chang-Gung Memorial Hospital in Taiwan. Patients who received IVI for AMD were recruited consecutively. The excluding criteria were not having a smartphone, not using the LINE app, being illiterate or unable to communicate in Mandarin, or being unwilling to use the PCCS.

The PCCS app was developed using the program code released from the LINE app (LINE Corporation) for both Android and iOS. Patients using the LINE app could use the PCCS app by scanning a QR code, and their data were securely stored in a cloud database. During the study, the app was securely linked with the data server of the cloud computer server in accordance with privacy protection standards mandated by the Taiwan Personal Data Protection Law.

Patients undergoing IVI and having a smartphone with an Android or iOS operating system and the LINE app installed were considered eligible. Patients were asked to participate in this study during their visit to the outpatient presurgery clinic based on their consent. In addition, the researcher helped the participants to use the app on their smartphone and provided verbal and “hands on” instructions on its usage and the course of the study. In brief, after downloading the LINE app from Google Play or App store, the patients were asked to allow the app to store data and access messages and contacts upon opening the app. Thereafter, the patients had to register an account by inputting their name and joining the PCCS group by scanning a QR code. Upon joining the PCCS group, they were required to input the date of IVI and select their ophthalmologist’s name. Patients’ account data were linked with the cloud database.

**Figure 1:**
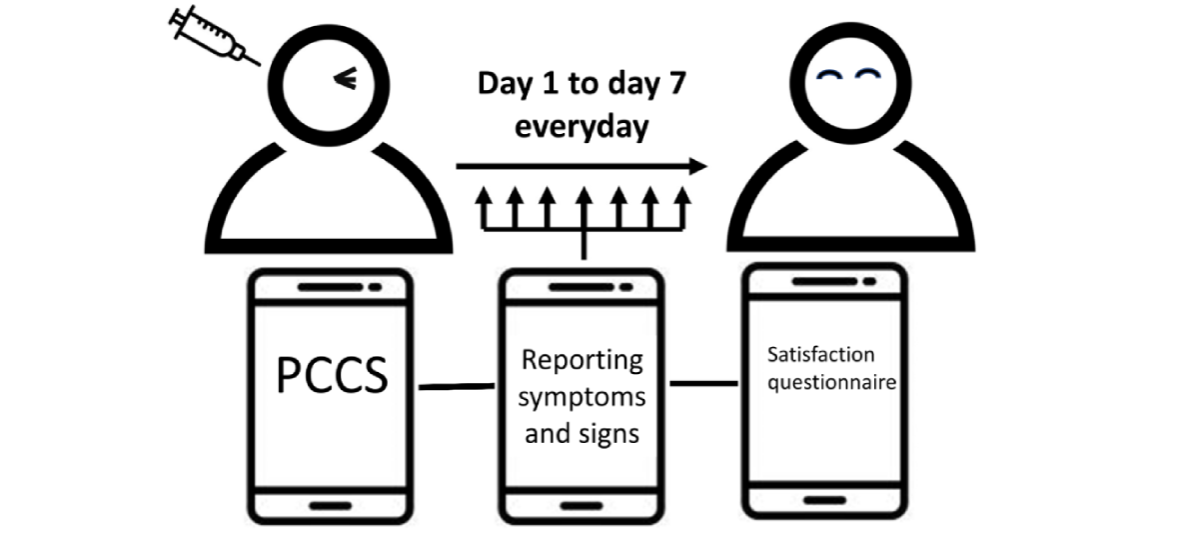
Patients used the postoperative chatbot system daily for 7 days following intravitreous injection to assess their symptoms and signs after receiving an alert. Satisfaction questionnaires were administered on day 7. PCCS: postoperative care chatbot system.

**Figure 2:**
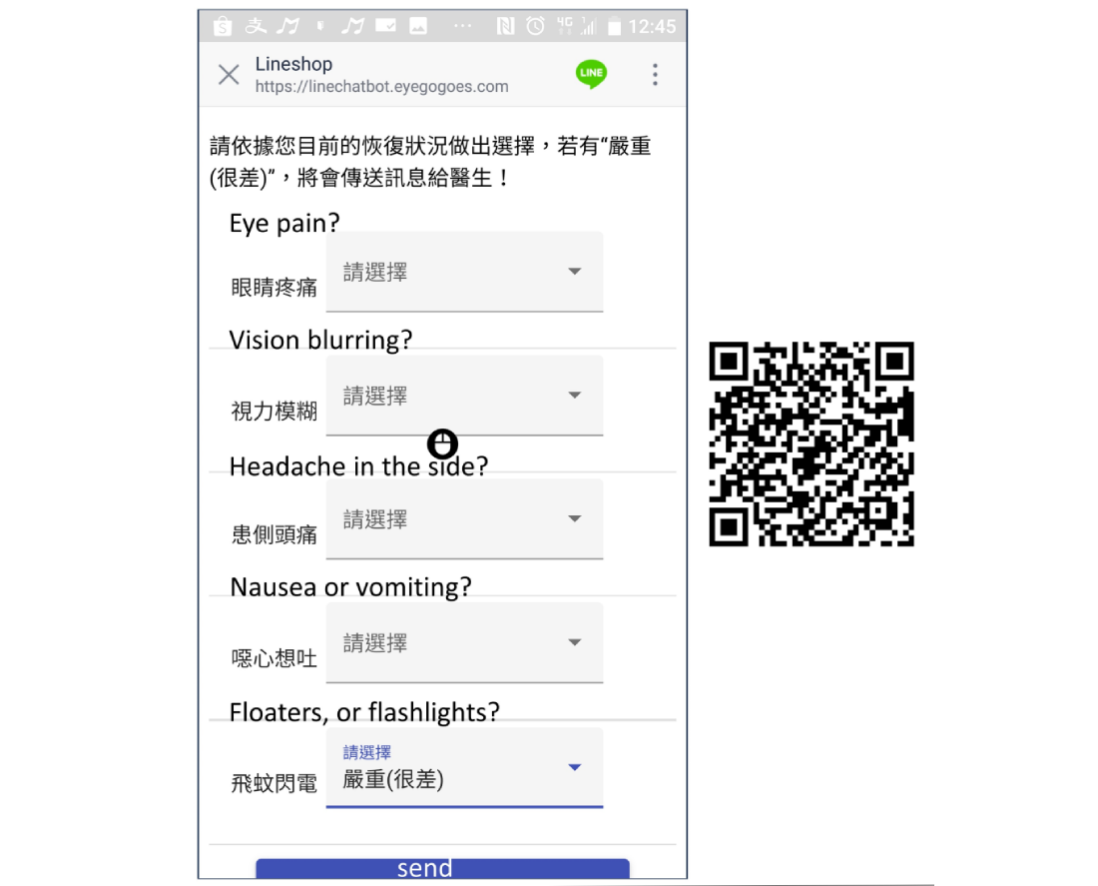
The prototypical interface of the postoperative care chatbot system accessed on a smartphone by scanning a QR code. All patients received a daily notification, consisting of 6 symptom- and sign-related questions, and were requested to provide answers regarding their condition.

Once the patients had left the postoperative care room after receiving IVI, they were able start using the app to self-report any postoperative symptoms and signs (Figure 1). In addition, all patients received a notification every day and six questions (Figure 2) about their condition: (1) Eye pain? (2) Eye redness/swelling? (3) Vision blurring? (4) Headache in the side? (5) Nausea or vomiting? (6) Floaters, or flashlights? Each question had four potential answers: (1) Better, (2) Same or none, (3) Not good, and (4) Worse. If the patient chose the “Worse” option in any question, the app would send a notification to the medical facility to decide whether a face-to-face examination in the outpatient department (OPD) or emergency room (ER) was required. A questionnaire regarding satisfaction was automatically presented in the PCCS app on the end of the seventh day post surgery to collect the patients’ feedback, which included the following three questions: (1) Do you feel satisfied with using the PCCS to report ocular symptoms instantly? (2) Do you think that the PCCS is helpful to improve the quality of care? (3) Are you willing to use the PCCS again in the future? Each of these questions had three response options for patients to choose: (1) Satisfied, (2) No comment, and (3) Unsatisfied.

### Ethical Considerations

The study was approved by the institutional review boards of Chang Gung Memorial Hospital (202201604B0). Written informed consent was obtained from all participants.

## Results

A total of 185 patients (87 women and 98 men) used the PCCS app after IVI. Among them, 53% (n=185) answered the app questions by themselves, 34% had their children’s respond, and 13% had others respond.

A total of 858 responses were obtained from the patients. There were 26 (3.0%) reports among 12 (6.5%) patients who chose the “Worse” option once during the 7-day follow-up. Deterioration was reported 2.16 (range 1-7; 26 divided by 12) times on average per patient, and there was no significant difference in the gender of these patients (chi-square *P*=.83). Six patients reported deterioration of blurred vision, 3 with increased floaters or photopsia, 2 with eye swelling or redness, and 1 with nausea or vomiting. Messages were sent to the medical staff, and the patients were contacted by phone for further OPD or ER visits. Seven of 12 patients returned to the OPD earlier after having been suggested to do so during the phone consultation. One patient with a history of diabetes mellitus reported deterioration of blurred vision and was diagnosed with endophthalmitis. Ocular inflammation and infection subsided after prompt IVI of antibiotics. Seven of the 185 (3.8%) patients had an earlier medical consultation and 1 (0.5%) had endophthalmitis.

The post–PCCS app use survey over 7 days shows that 87% (n=185) of the patients were satisfied with the use of the PCCS app to communicate their symptoms instantly; most felt that it was helpful to improve the quality of post-IVI care and were willing to use it again in the future.

## Discussion

In this study, the chatbot system detected 4% of patients needing earlier medical consultation without gender differences, of whom 0.5% required urgent management within 7 days post IVI. Patients reported being satisfied with the app’s enhancement of the quality of care.

Large retrospective studies have reported that around 0.6%-1.9% of patients seek urgent or unscheduled visits within 1-2 weeks after IVI, which is lower than what our study reports [8,11]. Gender differences in seeking medical consultation have been reported [12]. The chatbot system may enhance detection and eliminate gender differences.

Acute postoperative endophthalmitis frequently occurs within 1 week post surgery. Early detection and prompt management could possibly achieve better outcomes [7,13]. In contrast, a treatment delay may lead to irreversible vision loss. In this study, due to early report of deteriorated blurred vision from a patient, prompt medical intervention was provided, and the patient’s vision was salvaged after emergency treatment.

A new anti-VEGF antibody, brolucizumab, has reportedly shown adverse reactions in intraocular inflammation (IOI), such as retinal vasculitis and retinal vascular occlusion [14]. IOI occurrence should be alerted in patients treated with brolucizumab 6 mg (HAWK study: 5.3%; HARRIER study: 2.7%). Therefore, more frequent follow-up and prompt treatment are suggested for those receiving IVI of brolucizumab. The PCCS app might be a suitable choice for follow-up to promptly report the possibility of IOI after IVI of brolucizumab.

This chatbot system could thus be considered a supplementary telehealth option for postoperative ophthalmologist-delivered care to help monitor patients remotely. With the assistance of artificial intelligence and instant feedback without delay, physicians can better individualize the care of patients and treatment suggestions, and minimize clinic visits. It can also improve patient care and reduce costs after IVI, especially for those who live in rural areas. It can also be a potential solution in situations where fewer visits are possible, such as during the COVID-19 pandemic to minimize potential exposure to SARS-CoV-2. Furthermore, this PCCS app is potentially applicable in any kind of surgery to offer holistic care to patients. It could also contribute to improvement of the doctor-patient relationship.

This study has some limitations including a small sample, its retrospective nature, and the lack of a control group. Some older people or those with poor vision would be unable to use a smartphone and may need a family member’s assistance.

This smartphone-based chatbot system designed for self-reporting postoperative symptoms and providing instant bidirectional feedback could be beneficial for patients with AMD, enhancing early medical consultation without gender differences. It achieved satisfactory responses from the patients. It can potentially improve the quality of patient care after IVI and may be applicable to other surgical procedures.

## Abbreviations

AMD: age-related macular degeneration
ER: emergency room
IOI: intraocular inflammation
IVI: intravitreal injection
OPD: outpatient department
PCCS: postoperative care chatbot system
VEGF: vascular endothelial growth factor

## Data Availability

The data are available from the corresponding author upon reasonable request.

## References

[1] Pascolini D, Mariotti S, Pokharel G, Pararajasegaram R, Etya'ale D, Négrel A D, Resnikoff S (2004). 2002 global update of available data on visual impairment: a compilation of population-based prevalence studies. Ophthalmic Epidemiol.

[2] Yu J, Ba J, Peng R, Xu D, Li Y, Shi H, Wang Q (2015). Intravitreal anti-VEGF injections for treating wet age-related macular degeneration: a systematic review and meta-analysis. DDDT.

[3] Lanzetta P, Mitchell P, Wolf S, Veritti D (2013). Different antivascular endothelial growth factor treatments and regimens and their outcomes in neovascular age-related macular degeneration: a literature review. Br J Ophthalmol.

[4] Silva R, Berta A, Larsen M, Macfadden W, Feller C, Monés Jordi, TREND Study Group (2018). Treat-and-extend versus monthly regimen in neovascular age-related macular degeneration: results with ranibizumab from the TREND study. Ophthalmology.

[5] Gonzalez-Gonzalez LA, Knickelbein JE, Doft BH, Balasubramani GK, Wisniewski S (2023). Incidence and visual outcomes of acute endophthalmitis post intravitreal injection of anti-vascular endothelial growth factors in a single referral center. Int Ophthalmol.

[6] Singh R, Davoudi S, Ness S (2022). Preventive factors, diagnosis, and management of injection-related endophthalmitis: a literature review. Graefes Arch Clin Exp Ophthalmol.

[7] Dave V, Joseph J, Pathengay A, Pappuru R, Das T (2020). Clinical presentations, diagnosis, and management outcomes of Curvularia endophthalmitis and a review of literature. Retina.

[8] Miller A, Wilneff MA, Yazji A, Petrinec E, Carbone M, Miller C, McCrossin C, Donkor R, Miller DG (2022). Analysis of urgent follow up visits and complications after intravitreal injections: a retrospective cohort study. Int J Retina Vitreous.

[9] Chaikitmongkol Voraporn, Sagong Min, Lai Timothy Y Y, Tan Gavin S W, Ngah Nor Fariza, Ohji Masahito, Mitchell Paul, Yang Chang-Hao, Ruamviboonsuk Paisan, Wong Ian, Sakamoto Taiji, Rajendran Anand, Chen Youxin, Lam Dennis S C, Lai Chi-Chun, Wong Tien Yin, Cheung Chui Ming Gemmy, Chang Andrew, Koh Adrian (2021). Treat-and-extend regimens for the management of neovascular age-related macular degeneration and polypoidal choroidal vasculopathy: consensus and recommendations from the Asia-Pacific Vitreo-retina Society. Asia Pac J Ophthalmol (Phila).

[10] Borrelli E, Grosso D, Vella G, Sacconi R, Battista M, Querques L, Zucchiatti I, Prascina F, Bandello F, Querques G (2020). Short-term outcomes of patients with neovascular exudative AMD: the effect of COVID-19 pandemic. Graefes Arch Clin Exp Ophthalmol.

[11] Ramos MS, Xu LT, Singuri S, Castillo Tafur JC, Arepalli S, Ehlers JP, Kaiser PK, Singh RP, Rachitskaya AV, Srivastava SK, Sears JE, Schachat AP, Babiuch AS, Sharma S, Martin DF, Lowder CY, Singh AD, Yuan A, Nowacki AS (2021). Patient-reported complications after intravitreal injection and their predictive factors. Ophthalmol Retina.

[12] Wang Y, Hunt K, Nazareth I, Freemantle N, Petersen I (2013). Do men consult less than women? An analysis of routinely collected UK general practice data. BMJ Open.

[13] Banker A S, Freeman W R (2001). Retinal detachment. Ophthalmol Clin North Am.

[14] Holz F, Iida T, Maruko I, Sadda S (2022). A consensus on risk mitigation for brolucizumab in neovascular age-related macular degeneration: patient selection, evaluation, and treatment. Retina.

